# Multi-task learning identifies shared genetic risk for late-onset epilepsy and alzheimer’s disease

**DOI:** 10.1038/s41598-025-32329-8

**Published:** 2025-12-13

**Authors:** Mingzhou Fu, Thai Tran, Bogdan Pasaniuc, Keith Vossel, Timothy S. Chang

**Affiliations:** 1 Mary S. Easton Center for Alzheimer’s Research and Care, Department of Neurology, David Geffen School of Medicine, University of California, Los Angeles, CA 90095 USA; 2 Medical Informatics Home Area, Department of Bioinformatics, University of California, Los Angeles, CA 90095 USA; 3 Genomics and Computational Biology, University of Pennsylvania, Philadelphia, PA 19104 USA

**Keywords:** Alzheimer’s disease, Late-onset epilepsy, Shared genetic risks, Multi-task learning, Electronic health record, Computational biology and bioinformatics, Genetics, Neurology

## Abstract

Aging populations face increasing incidence of neurological disorders, including Alzheimer’s disease (AD) and late-onset epilepsy (LOE), which demonstrate a bidirectional relationship where AD is a risk factor for LOE and LOE is a risk factor for AD. While the *APOE* gene is a known shared risk factor, comprehensive genetic studies for LOE remain limited. This study employed a multi-task learning framework using Elastic Net modeling to systematically identify shared genetic risk factors between AD and LOE. We analyzed electronic health records from UCLA Health System (*N* = 416,212; genetic subset *N* = 16,500) and validated findings in the All of Us dataset (*N* = 52,493). Longitudinal analyses confirmed strong bidirectional associations between AD and LOE. The multi-task learning approach identified eight shared-risk single nucleotide polymorphisms mapping to key genes including the *APOE-TOMM40-APOC1* cluster, *BIN1*, *CLU*, *PVRL2*, and *TRAPPC6A*. These shared-risk genes were enriched in pathways related to lipid metabolism, amyloid catabolic processes, and tau protein binding. A shared genetic risk score effectively stratified patients into distinct AD-LOE risk groups. This study represents an initial systematic identification of potential shared genetic factors between AD and LOE using multi-task learning. While our findings suggest possible shared genetic contributions, particularly in the *APOE* region, and highlight tau-mediated mechanisms as potential therapeutic targets, further validation is needed to establish the extent of genetic overlap between these conditions.

**Supplementary Information:**

The online version contains supplementary material available at 10.1038/s41598-025-32329-8.

## Introduction

Aging represents a global demographic phenomenon that poses significant challenges to healthcare systems and societies worldwide. As populations age, the incidence of age-related neurological disorders increases substantially, including Alzheimer’s disease (AD) and late-onset epilepsy (LOE). AD is a progressive neurodegenerative disease that impairs memory, cognition, and the ability to perform activities of daily living. It is the leading cause of dementia, accounting for approximately 60–80% of all dementia cases^1^. Currently, over six million Americans live with AD, and one in three seniors dies with AD or related dementia^2^. The economic burden of caring for individuals with AD is projected to reach $1 trillion annually in the United States by 2050^2^. LOE, characterized by recurrent unprovoked seizures with onset at age 60 or older, also becomes increasingly prevalent with advancing age, with an incidence estimated at 80 to 102 cases per 100,000 people over 60 years of age^3–5^.

Extensive research from animal models to large epidemiologic studies has established a bidirectional relationship between AD and LOE. Individuals with AD demonstrate a two- to four-fold increased risk of developing epilepsy, with a significant proportion experiencing their first seizure concurrently with, or several years preceding, the onset of cognitive decline^6^. Conversely, LOE is associated with a two- to three-fold increased risk of developing dementia^7^. Clinical studies vary widely but report that around 20% of AD patients experience seizures during follow-up periods, with seizure onset occurring at any stage of disease progression^8,9^. Supporting this clinical evidence, many mouse models of AD develop seizures and epileptiform spiking in early stages of the disease, prior to the deposition of disease-related proteins and onset of cognitive decline^10^. Moreover, patients with clinical and subclinical epileptiform activity tend to develop AD at younger ages and experience more rapid cognitive decline compared to those without epilepsy^11,12^. An emerging hypothesis proposes the existence of an epileptic prodromal variant of AD, in which development of LOE represents the first clinical symptom of AD in a subset of patients and serves as a harbinger for subsequent cognitive decline^13^. While this association may arise from multiple factors including shared pathophysiological mechanisms, neuroinflammation, and structural brain changes^14^, the present study focuses specifically on the genetic underpinnings of this relationship.

While large-scale genome-wide association studies (GWASs) have successfully identified numerous common genetic risk factors for AD, including the prominent apolipoprotein E (*APOE*) gene^15^, there are no GWAS studies for LOE. This gap in the literature limits the ability to examine shared genetic risk factors between AD and LOE using established cross-trait meta-analysis methods such as conjunctional false discovery rate^16^ or cross-trait linkage disequilibrium score regression (LDSC)^17^, which are designed to estimate genetic correlations and identify shared loci between related conditions.

To date, shared genetic risk factors between AD and LOE have been identified primarily through observational studies, with *APOE* serving as the most extensively characterized. Carriers of the *APOE-ε4* allele demonstrate a 1.4–2.1-fold increased risk of developing LOE compared to non-carriers^18,19^. Additionally, *APOE-ε4* allele carriers exhibit greater susceptibility to postictal confusion^20^. However, a comprehensive characterization of the shared genetic architecture between AD and LOE, including both the well-established *APOE* region and additional genetic factors, remains lacking. The extent to which genetic overlap extends beyond *APOE* and the biological mechanisms underlying such shared genetic susceptibility require systematic investigation.

This study aims to comprehensively characterize the shared genetic architecture between AD and LOE, including both established factors, such as *APOE* and novel genetic variants, and to elucidate the biological pathways that may link these genetic variants to the development of both conditions. The findings may enhance our understanding of the disease mechanisms underlying AD and LOE, potentially informing the development of targeted prevention and treatment strategies for these neurological disorders. Furthermore, the methodological approaches employed in this study could be applied to other complex diseases to evaluate shared genetic risks, offering valuable insights into potential causal mechanisms and pathways.

## Results

### UCLA sample description

**Fig. 1:**
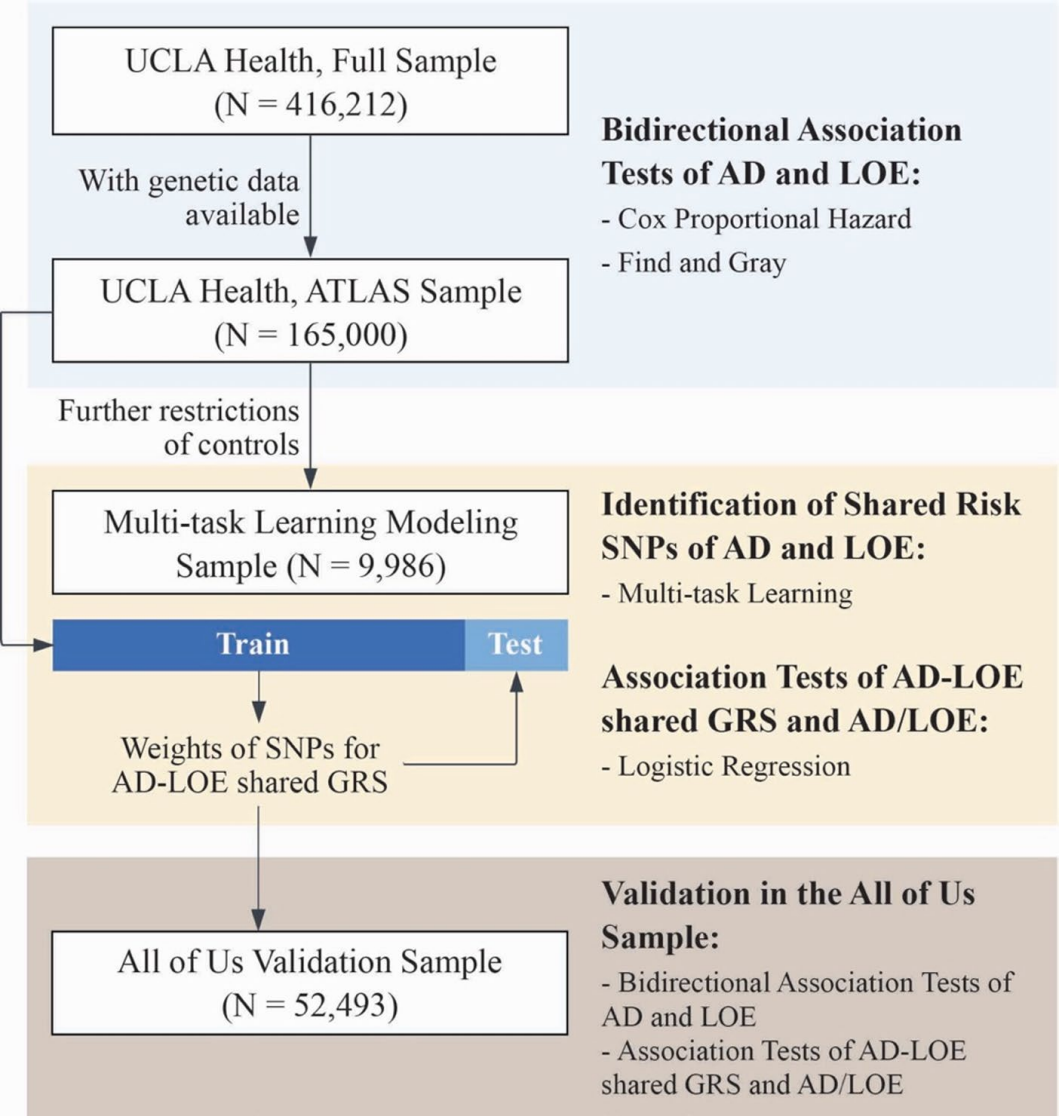
Overview of study design and samples included in the analyses.

Figure 1 provides an overview of the study design and the samples included in the analyses. The primary analytical sample was derived from the electronic health records (EHRs) of the University of California, Los Angeles (UCLA) Health System, specifically from individuals who self-reported as non-Hispanic Whites. Table 1 presents baseline descriptive statistics for our full UCLA sample (*N* = 416,212). In this cohort, we observed an older age at last visit, a longer span of EHR records, and a higher proportion of deceased patients in LOE or AD cases compared to their corresponding controls. Additionally, there was a higher prevalence of hypertension, diabetes, stroke, and hyperlipidemia among individuals with LOE or AD compared to controls. The proportion of patients with complications related to AD or LOE was also higher compared to controls.

**Table 1:** Descriptive statistics of the analytical sample by late-onset epilepsy and Alzheimer’s disease status, full UCLA patient population (*N* = 416,212)^a^.

	Late-onset epilepsy			Alzheimer’s disease		
	Cases	Controls	*P*-value	Cases	Controls	*P*-value
	*N* = 7,351	*N* = 408,861		*N* = 6,706	*N* = 409,506	
Primary outcome(s)						
Alzheimer’s disease (Yes)	571 (7.8%)	6,135 (1.5%)	< 0.001*	6,706 (100%)	0 (0%)	-
Late onset epilepsy (Yes)	7,351 (100%)	0(0%)	-	571 (8.5%)	6,780 (1.7%)	< 0.001*
Demographics						
Age at first visit (in years)	60.89 (11.44)	60.10 (11.32)	< 0.001*	65.75 (11.38)	60.02 (11.30)	< 0.001*
Age at last visit (in years)	75.76 (7.37)	72.30 (7.87)	< 0.001*	81.68 (5.83)	72.21 (7.81)	< 0.001*
Sex (female)	3,718 (51%)	221,290 (54%)	< 0.001*	4,060 (61%)	220,948 (54%)	< 0.001*
EHR record length (in years)	14.87 (10.72)	12.20 (9.84)	< 0.001*	15.93 (10.89)	12.19 (9.83)	< 0.001*
Deceased (Yes)	1,336 (18%)	18,391 (4.5%)	< 0.001*	1,601 (24%)	18,126 (4.4%)	< 0.001*
Health conditions						
Hypertension (Yes)	5,031 (68%)	143,043 (35%)	< 0.001*	4,592 (68%)	143,482 (35%)	< 0.001*
Diabetes (Yes)	1,624 (22%)	43,392 (11%)	< 0.001*	1,323 (20%)	43,693 (11%)	< 0.001*
Stroke (Yes)	345 (4.7%)	2,495 (0.6%)	< 0.001*	572 (8.5%)	2,268 (0.6%)	< 0.001*
Hyperlipidemia (Yes)	4,128 (56%)	122,184 (30%)	< 0.001*	3,864 (58%)	122,448 (30%)	< 0.001*

*EHR* electronic health record.

[a] Summary statistics are displayed as n (%) for categorical variables, and mean (SD) for continuous variables. P-values were calculated with Pearson’s Chi-squared test or Wilcoxon rank sum test as appropriate.

*Statistical significance at P-value < 0.05 level.

The secondary analytical sample was derived from a subset of the primary sample, comprising patients with available genetic data (ATLAS sample, *N* = 16,500). Compared to patients without genetic data (non-ATLAS sample, *N* = 399,712), our ATLAS sample exhibited a higher prevalence of AD and LOE, were younger at first visit but older at last visit, had longer EHR record lengths, and a higher rate of deceased patients. The ATLAS sample also presented with worse baseline health conditions, indicated by a higher prevalence of hypertension, diabetes, stroke, and hyperlipidemia (Supplementary Table 1).

Table 2 presents baseline descriptive statistics for our UCLA ATLAS sample. In this sample, similar patterns of distributions were observed across AD/LOE cases, demographics, and health conditions. The proportion of patients with complications related to AD or LOE was higher in cases compared to controls. The distribution of *APOE-ε4* allele count and AD polygenic risk score (PRS) without the *APOE* region also differed significantly between AD cases and controls, with a higher prevalence in AD cases. However, there were no significant differences in *APOE-ε4* allele count or AD PRS without the *APOE* region distributions between LOE cases and controls.

**Table 2:** Descriptive statistics of the analytical sample by late-onset epilepsy and Alzheimer’s disease (AD) status, UCLA ATLAS patient population (*N* = 16,500)^a^.

	Late-onset epilepsy			Alzheimer’s disease		
	Cases	Controls	*P*-value	Cases	Controls	*P*-value
	*N* = 658	*N* = 15,842		*N* = 376	*N* = 16,124	
Primary outcome(s)						
Alzheimer’s disease (yes)	44 (6.7%)	332 (2.1%)	< 0.001*	376 (100%)	0 (0%)	-
Late onset epilepsy (yes)	658 (100%)	0 (0%)	-	44 (12%)	614 (3.8%)	< 0.001*
Demographics						
Age at first visit (in years)	58.19 (10.36)	56.15 (11.60)	< 0.001*	62.08 (11.83)	56.09 (11.52)	< 0.001*
Age at last visit (in years)	75.95 (6.77)	73.26 (7.59)	< 0.001*	82.01 (5.93)	73.16 (7.49)	< 0.001*
Sex (female)	298 (45%)	7,794 (49%)	0.049*	207 (55%)	7,885 (49%)	0.02*
EHR record length (in years)	17.76 (9.81)	17.11 (10.24)	0.03*	19.93 (10.92)	17.07 (10.20)	< 0.001*
Deceased (yes)	141 (21%)	1,221 (7.7%)	< 0.001*	67 (18%)	1,295 (8.0%)	< 0.001*
Health conditions						
Hypertension (yes)	526 (80%)	10,849 (68%)	< 0.001*	296 (79%)	11,079 (69%)	< 0.001*
Diabetes (yes)	188 (29%)	3,516 (22%)	< 0.001*	103 (27%)	3,601 (22%)	0.02*
Stroke (yes)	48 (7.3%)	315 (2.0%)	< 0.001*	54 (14%)	309 (1.9%)	< 0.001*
Hyperlipidemia (yes)	524 (80%)	11,589 (73%)	< 0.001*	304 (81%)	11,809 (73%)	< 0.001*
AD genetics						
* APOE-ε4* count			0.5			< 0.001*
0	483 (73%)	11,932 (75%)		211 (56%)	12,204 (76%)	
1	167 (25%)	3,707 (23%)		142 (38%)	3,732 (23%)	
2	8 (1.2%)	203 (1.3%)		23 (6.1%)	188 (1.2%)	
AD PRS with *APOE* region	0.11 (1.23)	0.06 (1.18)	0.2	0.28 (1.21)	0.05 (1.18)	< 0.001*

*APOE* apolipoprotein E, *EHR* electronic health record.

[a] Summary statistics are displayed as n (%) for categorical variables, and mean (SD) for continuous variables. P-values were calculated with Pearson’s Chi-squared test or Wilcoxon rank sum test as appropriate.

*Statistical significance at P-value < 0.05 level.

### Associations between LOE and AD phenotypes

We employed two longitudinal models to assess the associations between LOE and AD phenotypes in the full UCLA sample (*N* = 416,212) and the UCLA ATLAS sample (*N* = 16,500): the Cox proportional hazard model^21^ and the Fine and Gray model^22^. The association results are presented in Table 3.

**Table 3:** Associations between Alzheimer’s disease (AD) and late-onset epilepsy (LOE), UCLA patient population^a^.

	AD ~ LOE			LOE ~ AD		
	*N*	HR [95% CI]	*P*-value	*N*	HR [95% CI]	*P*-value
UCLA full sample (*N* = 416,212)						
Basic model^a^						
Cox proportional hazard	415,623	6.86 (6.02, 7.81)	< 0.001*	415,364	6.31 (5.49, 7.25)	< 0.001*
Fine and Gray	415,623	2.54 (2.12, 3.06)	< 0.001*	415,364	2.13 (1.36, 3.34)	0.001*
UCLA ATLAS sample (*N* = 16,500)						
Basic model^a^						
Cox proportional hazard	16,480	10.0 (6.43, 15.6)	< 0.001*	16,462	6.71 (4.06, 11.1)	< 0.001*
Fine and Gray	16,480	3.55 (2.27, 5.55)	< 0.001*	16,462	2.26 (1.31, 3.88)	0.003*
Adjusted for AD genetics^b^						
Cox proportional hazard	16,480	10.2 (6.53, 15.9)	< 0.001*	16,462	6.55 (3.96, 10.9)	< 0.001*
Fine and gray	16,480	3.77 (2.43, 5.83)	< 0.001*	16,462	2.20 (1.28, 3.81)	0.005*

*CI* confidence interval, *EHR* electronic health record, *HR* hazard ratio.

[a]Basic Model adjustments include age at first visit, sex, EHR record length, hypertension, diabetes, stroke, and hyperlipidemia status.

[b]Additionally adjusted for *APOE-ε4* count and AD polygenic risk score (without *APOE* region) in [b].

In the full UCLA sample, for the associations between LOE and AD, where incidence of AD was considered the outcome, after adjusting for age at first visit, sex, EHR record length, and health risk factors common to both conditions (including hypertension, diabetes, stroke, and hyperlipidemia), the Cox proportional hazard model demonstrated a strong positive association between LOE and the incidence of AD (Hazard Ratio (HR) = 6.86, 95% confidence interval (CI): 6.02, 7.81). This association still held after accounting for the competing risk of death (HR = 2.54, 95% CI: 2.12, 3.06). Regarding associations between AD and LOE, where LOE was the outcome, a stronger positive association was observed between AD status and the incidence of LOE in the Cox proportional hazard model (HR = 6.31, 95% CI: 5.49, 7.25). This association still held after accounting for the competing risk of death (HR = 2.13, 95% CI: 1.36, 3.34).

In the ATLAS sample, we observed similar increased risks of AD in LOE patients and vice versa, even after accounting for the competing risk of death. When additionally adjusted for AD genetic factors, including the *APOE-ε4* allele count and AD polygenic risk score (without the *APOE* region), these positive associations persisted. Further examination of the effect sizes of these genetic factors (Supplementary Table 2) revealed that only *APOE-ε4* allele count was significantly associated with an increased risk of AD when adjusting for LOE status (Cox model: HR = 2.53, 95% CI: 2.10, 3.05; Fine and Gray model: HR = 2.51, 95% CI: 2.07, 3.05). There was a suggestive positive association between *APOE-ε4* allele count and the incidence of LOE when adjusting for AD status, but it did not reach statistical significance. No statistically significant association was found between the AD polygenic risk score (without the *APOE* region) and either AD or LOE.

These association results suggest that *APOE* may be a significant risk factor for both conditions and could play a role in their co-occurrence or complications. However, a crucial question remains: are there shared risk factors beyond *APOE* that are not captured by the AD PRS without the *APOE* region? Investigating their shared genetic risk is essential. Understanding potential genetic overlap could provide insights into underlying biological mechanisms that these models may not fully capture, ultimately contributing to a more comprehensive understanding of the relationship between AD and LOE.

Genetic correlation with LDSC regression.

We initially assessed the genetic correlations between AD and generalized epilepsy using the LDSC regression method with publicly available GWAS summary statistics^23,24^. Due to the absence of GWAS data specific to LOE, we utilized data from a recent GWAS on generalized epilepsy^23^. The LDSC analysis revealed no significant genetic correlation between AD and generalized epilepsy (*rg* = -0.10 ± 0.06, *p* = 0.10).

Although LDSC regression identified a null association between AD and epilepsy, this result may reflect mechanistic differences in generalized epilepsy and LOE, and certain limitations inherent to the LDSC approach. LDSC is constrained by its reliance on GWAS summary statistics and may lack the power to detect complex genetic relationships or interactions that influence both AD and LOE^17^. To overcome these limitations, we turned to a multi-task machine learning model, which can simultaneously evaluate multiple outcomes, incorporate more intricate genetic architectures, and potentially uncover shared risk factors that traditional methods might miss^25^. This approach offers a more nuanced exploration of the genetic underpinnings of AD and LOE.

Shared genetic risks of AD and LOE with multi-task learning.

**Fig. 2:**
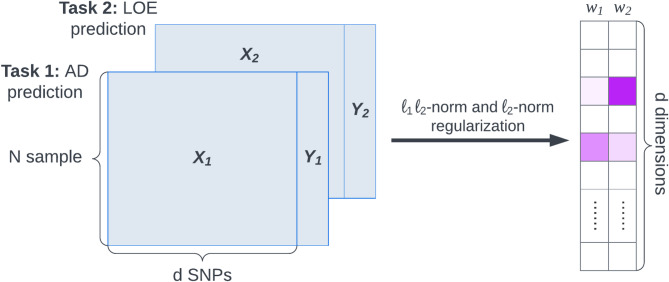
Multi-task elastic net model framework.

We applied a multi-task learning framework (Fig. 2, detailed in Methods) to identify shared risk single nucleotide polymorphisms (SNPs) for AD and LOE using a subset of the UCLA ATLAS sample (*N* = 9,986, including 376 AD cases and 658 LOE cases). In this subsample, we observed similar patterns to the full ATLAS sample that AD and LOE cases had a higher prevalence of condition-specific complications compared to controls. However, distributions of baseline demographics, EHR characteristics, and comorbidity profiles were different from those in the full ATLAS cohort due to stricter control criteria: age at last visit ≥ 70 and a minimum of five years of records. These requirements minimized misclassification bias and improved case–control balance (see Methods, Supplementary Table 3).

After accounting for age at last visit, sex, and ancestry-specific genetic variations (represented by principal components (PCs)), *APOE-ε4* was significantly associated with AD (OR = 2.85, 95% CI: 2.34, 3.48) and showed a borderline association with LOE (OR = 1.20, 95% CI: 0.99, 1.45), suggesting a modest shared genetic contribution. The AD polygenic risk score (without the *APOE* region) was associated with AD (OR = 1.15, 95% CI: 1.04, 1.27) but not with LOE (Table 4).

**Table 4:** Associations between Alzheimer’s disease (AD) genetic risk factors and AD or late-onset epilepsy (LOE), multi-task elastic net modeling sample (subset of UCLA ATLAS patient) (*N* = 9,986)^a^.

Outcome	*N*	APOE-ε4 count		AD PRS without APOE region	
		OR [95% CI]	*P*-value	OR [95% CI]	*P*-value
AD	9,986	2.85 (2.34, 3.48)	< 0.001*	1.15 (1.04, 1.27)	0.01*
LOE	9,986	1.20 (0.99, 1.45)	0.053	1.03 (0.95, 1.12)	0.52

*AD* Alzheimer’s disease, *CI* confidence interval.

[a]Basic Model adjustments include age at last visit, sex, and ancestry-specific principal components.

**Fig. 3:**
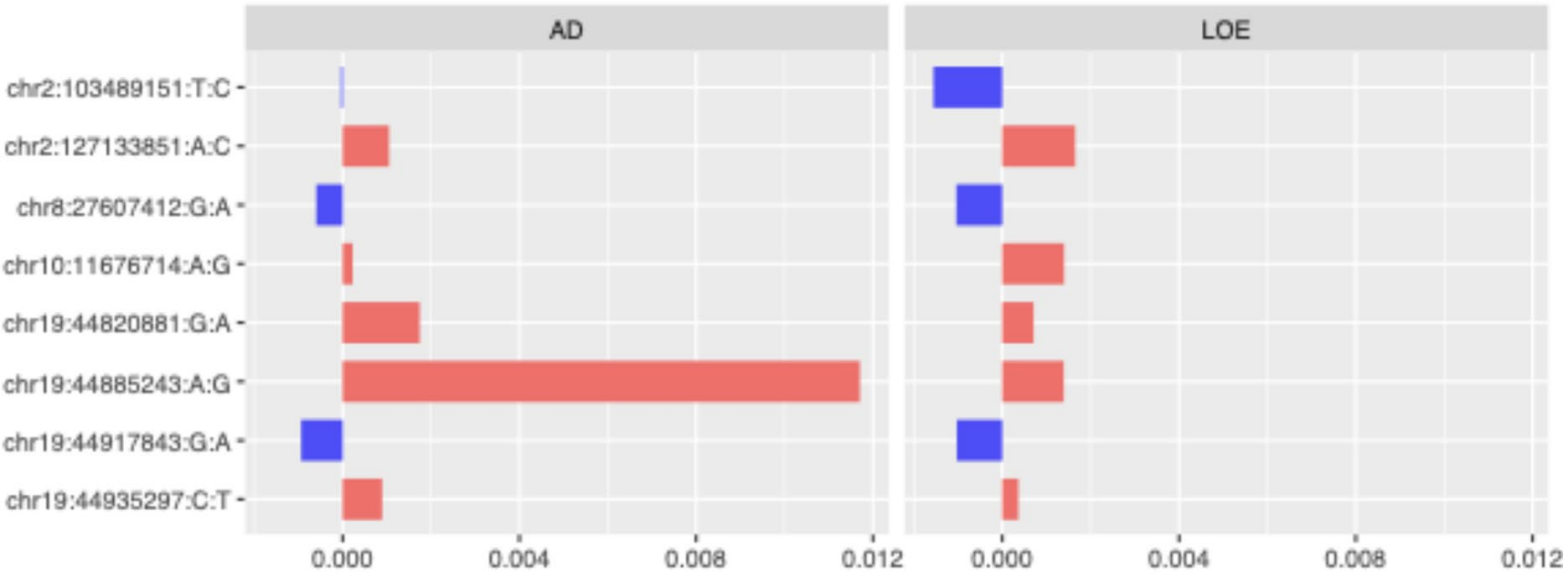
Standardized coefficient magnitude of shared risk features identified from the multi-task elastic net models. SNPs identified from the epilepsy GWAS are marked in orange. Colors in the bar plot represent direction of effects (red for ‘+’, blue for ‘-‘).

Using a multi-task Elastic Net model, we jointly predicted AD and LOE based on candidate SNPs from recent GWAS of AD^24^ and generalized epilepsy^23^ (see Methods) and incorporated offset corrections within a linearized framework. The model identified nine SNPs with non-zero weights for both phenotype prediction tasks. Among these, eight SNPs were identified as shared genetic risk factors for AD and LOE, defined by having coefficients in the same direction for both conditions. Seven of the eight were SNPs from the AD GWAS. The standardized coefficient magnitudes for these shared risk factors are presented in Fig. 3. A detailed list, including related genomic coordinates and functional annotations, were provided in Supplementary Table 4.

A set of shared risk SNPs was identified near the *19q13* region. Among these, chr19:44885243 was the top SNP with the highest coefficient magnitude, particularly in predicting AD. Additionally, SNPs outside the *19q13* region, such as chr2:127133851, chr8:27607412, and chr10:11676714, also contributed significantly to the shared genetic risk for AD and LOE, highlighting the complex genetic interplay between these disorders.

**Fig. 4:**
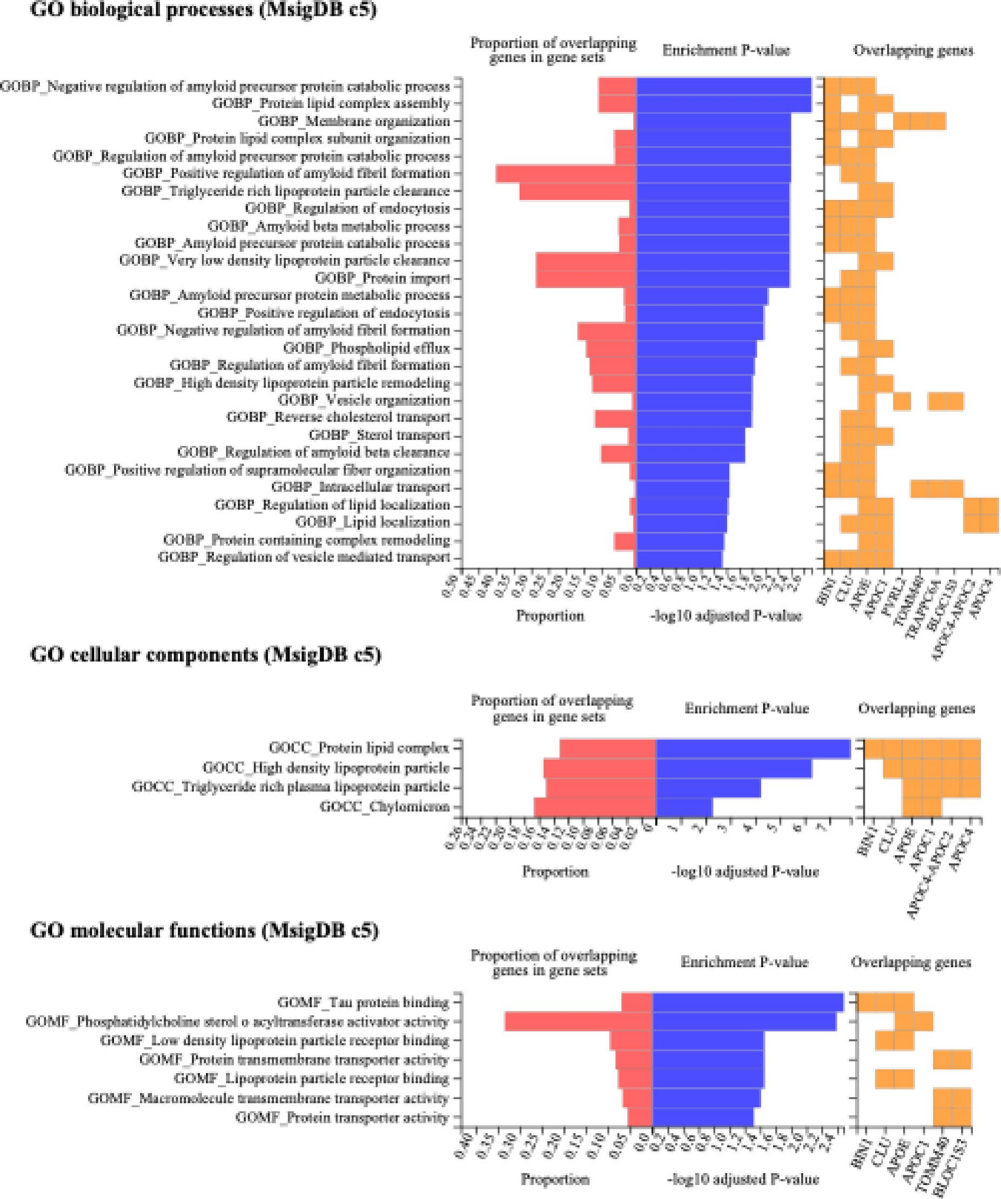
Enrichment test results for shared risk genes of Alzheimer’s disease and late-onset epilepsy. The set of genes were mapped from shared risk genetic loci from the multi-task Elastic Net model.

The eight shared risk SNPs were mapped to 21 genes using positional, expression quantitative trait loci (eQTL), and chromatin interaction mapping techniques. These genes were tested against established gene sets for overrepresentation in terms of positions, biological functions, and pathways (Fig. 4). The *APOE* region *19q13* (including the overlapping linkage disequilibrium (LD) block with *TOMM40* and *APOC1* genes), emerged as an essential genomic risk locus among the mapped shared risk genes for AD and LOE (Supplementary Table 5). Other important genes included *BIN1*, *CLU*, *PVRL2* and *TRAPPC6A*. The shared risk genes were enriched in gene ontology biological processes related to amyloid catabolic processes and lipid metabolism, cellular components such as protein lipid complex, lipoprotein particles, and chylomicron, and molecular functions including tau protein binding and phosphatidylcholine-sterol O-acyltransferase activity. Notably, the most significantly enriched pathways were primarily associated with AD pathogenesis, with limited evidence for epilepsy-specific pathways.

### Sensitivity analyses

#### Shared AD-LOE genetic risk score (GRS)

We calculated a shared AD-LOE GRS for each individual in the full UCLA ATLAS sample (*N* = 16,500) using the shared risk factors identified in the previous analysis. Weights for SNPs were estimated from a logistic regression model in the training set (*N* = 13,200, N AD = 540, N LOE = 312). The GRS was standardized to reflect per-unit increases in genetic risk. We then assessed the associations between this shared GRS and AD and/or LOE-related phenotypes in the testing set (*N* = 3,300, N AD = 64, N LOE = 118), comparing these results to the well-documented effect of the *APOE* gene (Table 5).

**Table 5:** Associations between shared Alzheimer’s disease (AD)-late-onset epilepsy (LOE) shared genetic risk score (GRS), APOE-ε4 count, and AD/LOE status, UCLA ATLAS patient population, with multi-task elastic net modeling testing sample (*N* = 2,002)^a^.

Phenotype	Variation explained	OR (95% CI)	*P*-value
APOE-ε4 count			
AD or LOE	1.44%	1.90 (1.41, 2.53)	< 0.001*
AD only	7.57%	4.31 (2.75, 6.78)	< 0.001*
LOE only	0.01%	1.05 (0.70, 1.54)	0.81
AD-LOE shared GRS (full)			
AD or LOE	1.79%	1.42 (1.22, 1.64)	< 0.001*
AD only	6.87%	2.10 (1.64, 2.71)	< 0.001*
LOE only	0.29%	1.16 (0.97, 1.38)	0.1
AD-LOE shared GRS (without APOE)			
AD or LOE	0.63%	1.24 (1.06, 1.45)	0.01*
AD only	0.72%	1.28 (0.99, 1.66)	0.056
LOE only	0.49%	1.21 (1.01, 1.46)	0.04*

*APOE* apolipoprotein E, *CI* confidence interval, *OR* odds ratio.

[a] Model adjustments include age at last visit, sex, and first five ancestry-specific principal components.

In the UCLA ATLAS multi-task modeling testing sample (*N* = 2,002), a one-standard deviation increase in the shared AD-LOE shared GRS was associated with a 1.42-fold increase in the risk of AD or LOE (95% CI: 1.22, 1.64); a 2.10-fold increase in the risk of AD (95% CI: 1.64, 2.71), and a borderline 1.16-fold increase in the risk of LOE (95% CI: 0.97, 1.38). The effect sizes were stronger when considering the *APOE-ε4* allele count in relation to AD or LOE (OR = 1.90, 95% CI: 1.41, 2.53) and AD (OR = 4.31, 95% CI: 2.75, 6.78). However, no association was observed between *APOE-ε4* allele count and LOE. While both *APOE*-ε4 and the shared GRS were associated with the combined AD or LOE phenotype, the associations appeared to be primarily driven by AD cases rather than showing equivalent effects across both conditions individually.

In terms of variation explained by the genetic scores, the *APOE-ε4* count explained a higher proportion of variance in AD-related phenotypes compared to the AD-LOE shared GRS. Conversely, the AD-LOE shared GRS explained a greater proportion of the variance in LOE-related phenotypes than the *APOE-ε4* count.

To address potential concerns about *APOE* region dominance in our shared genetic risk score, we conducted additional analyses focusing exclusively on the four shared risk variants located outside the *APOE* region (chr2:127133851, chr8:27607412, chr10:11676714, and chr2:103489151). These variants map to *BIN1*, *CLU*, *PVRL2*, and *TRAPPC6A* genes, respectively. The non-*APOE* shared genetic risk score demonstrated a significant association with the combined AD or LOE phenotype (OR = 1.24, 95% CI: 1.06, 1.45) and LOE-only (OR = 1.21, 95% CI: 1.01, 1.46), and a borderline associations with AD-only (OR = 1.28, 95% CI: 0.99, 1.66). These findings provide evidence for genetic risk sharing beyond the *APOE* region, with the non-*APOE* shared genetic risk score demonstrating significant associations with both the combined phenotype and LOE specifically.

#### Multi-task model performance in AD or LOE prediction

Although the primary aim of our study was to identify shared risk factors rather than to predict AD and LOE, we evaluated the performance of our multi-task Elastic Net model by comparing it with separate Elastic Net models that predicted AD and LOE individually with 1000 iterations (details in Methods). The multi-task Elastic Net model not only identified shared risk factors between AD and LOE but also contributed to the prediction of AD and LOE onset. When compared to the separate Elastic Net models, the multi-task Elastic Net model showed overall improvement in both AD and LOE prediction tasks. Specifically, we observed a significant improvement in the area under the precision-recall curve for AD prediction (0.083 vs. 0.061, Wilcoxon test p-value < 0.001) and a significant improvement for LOE prediction (0.108 vs. 0.078, Wilcoxon test p-value < 0.001).

#### Validation in all of Us dataset

To further assess robustness, we validated our results in a comparable non-Hispanic White sample extracted from the All of Us database (*N* = 52,493). Compared to the UCLA ATLAS sample (*N* = 16,500), the All of Us sample was older at their first visit and younger at their last visit on average, with a higher proportion of females, longer EHR record length, and a lower proportion of deceased patients. The All of Us sample also had a lower prevalence of AD but a higher prevalence of LOE, and better overall health conditions (Supplementary Table 6). Within the All of Us sample, we also observed similar patterns in distributions across AD/LOE cases, demographics, and health conditions. The proportion of patients with complications related to AD or LOE was higher in cases compared to controls (Supplementary Table 7).

First, we repeated the same association tests of AD and LOE with the Cox proportional hazard models and the Fine and Gray models in the All of Us sample. Results were shown in Table 6. After adjusting for age at first visit, sex, EHR record length, hypertension, diabetes, stroke, and hyperlipidemia, we observed a similar positive association between LOE and the incidence of AD (HR = 3.95, 95% CI: 2.77, 5.63). This association still held after accounting for the competing risk of death (HR = 2.47, 95% CI: 1.69, 3.60). Similarly, we also observed a positive association between AD and the incidence of LOE (Cox model: HR = 2.96, 95% CI: 1.89, 4.61; Fine and Gray model: HR = 1.98, 95% CI: 1.22, 3.30). After additionally adjusting for *APOE-ε4* count, the positive associations still held.

**Table 6:** Associations between Alzheimer’s disease (AD) and late-onset epilepsy (LOE), all of Us patient population (*N* = 52,493)^a^.

	AD ~ LOE			LOE ~ AD		
	*N*	HR [95% CI]	*P*-value	*N*	HR [95% CI]	*P*-value
Basic model^a^						
Cox proportional hazard	52,319	3.95 (2.77, 5.63)	< 0.001*	52,206	2.96 (1.89, 4.61)	< 0.001*
Fine and Gray	52,319	2.47 (1.69, 3.60)	< 0.001*	52,206	1.98 (1.22, 3.20)	0.005*
Adjusted for AD genetics^b^						
Cox proportional hazard	52,319	3.96 (2.78, 5.64)	< 0.001*	52,206	2.96 (1.89, 4.62)	< 0.001*
Fine and Gray	52,319	2.49 (1.71, 3.63)	< 0.001*	52,206	1.98 (1.22, 3.21)	0.001*

*CI* confidence interval, *EHR* electronic health record.

[a]Basic model adjustments include age at first visit, sex, EHR record length, hypertension, diabetes, stroke, and hyperlipidemia status.

[b] Additionally adjusted for *APOE-ε4* count and AD polygenic risk score (without *APOE* region) in [b].

We then applied the shared GRS weights, derived from the UCLA ATLAS training sample, to a subset of participants in the All of Us cohort with available genetic data. To ensure comparability, we restricted this evaluation sample using the same inclusion criteria as those applied to the UCLA genetic dataset. An individual-level AD–LOE shared GRS was subsequently computed for each participant in the All of Us subset (*N* = 23,037). We tested the associations between the AD-LOE shared GRS and both AD and LOE in this sample and compared them to the *APOE-ε4* model (Table 7). The positive associations still held between AD-LOE shared GRS and AD or LOE (OR = 1.09, 95% CI: 1.04, 1.14) and AD (OR = 1.54, 95% CI: 1.40, 1.69). However, the effect sizes were not as large as those in the UCLA ATLAS sample, where those weights were trained. The *APOE-ε4* allele count was associated with AD or LOE (OR = 1.16, 95% CI: 1.01, 1.32) and AD (OR = 1.98, 95% CI: 1.42, 2.84), with smaller effect sizes observed as well. The variation explained by the genetic scores was also lower for both variables compared to the UCLA ATLAS sample. However, the non-*APOE* shared genetic risk score did not show a statistically significant association with any of the tested phenotypes, including the combined AD or LOE outcome, AD-only, or LOE-only.

**Table 7:** Associations between shared Alzheimer’s disease (AD)-late-onset epilepsy (LOE) shared genetic risk score (GRS), APOE-ε4 count, and AD/LOE status, all of Us patient population, genetic sample (*N* = 23,037)^a^.

Phenotype	Variation explained	OR (95% CI)	*P*-value
APOE-ε4 count			
AD or LOE	0.04%	1.16 (1.01, 1.32)	0.03*
AD only	0.46%	1.98 (1.42, 2.84)	< 0.001*
LOE only	< 0.01%	1.04 (0.90, 1.20)	0.59
AD-LOE shared GRS (full)			
AD or LOE	0.10%	1.09 (1.04, 1.14)	< 0.001*
AD only	2.02%	1.54 (1.40, 1.69)	< 0.001*
LOE only	< 0.01%	1.01 (0.96, 1.06)	0.80
AD-LOE shared GRS (without APOE)			
AD or LOE	0.01%	1.04 (0.95, 1.14)	0.41
AD only	0.06%	1.17 (0.96, 1.42)	0.12
LOE only	0.02%	1.09 (0.98, 1.20)	0.11

*APOE* apolipoprotein E, *CI* confidence interval, *OR* odds ratio.

[a] Model adjustments include age at last visit, sex, and first five ancestry-specific principal components.

## Discussion

Using a multi-task Elastic Net method, we identified eight shared risk SNPs between AD and LOE, which were then mapped to genes for further biological function analysis. Our findings highlight the *APOE* region, including the overlapping LD block with *TOMM40* and *APOC1* genes, as a significant genomic risk locus for both conditions. Additionally, genes such as *BIN1*, *CLU*, *PVRL2*, and *TRAPPC6A* were identified as key shared risk factors. These genes are enriched in pathways related to lipid metabolism, amyloid catabolic processes, and tau protein binding. This approach enabled the discovery of common genetic factors, providing valuable insights into shared disease mechanisms that could inform prevention, targeted treatments, and drug development for both AD and LOE.

Few studies have evaluated LOE and AD associations with longitudinal models^26,27^, and none have further included genetic risk factors, such as the *APOE* gene and AD PRS, as covariates to control for genetic factors that could contribute to both conditions. To further explore the complex associations between LOE and AD, we employed two different longitudinal models (Cox proportional hazard and Fine and Gray models) in two different populations (UCLA Health and All of Us). Our findings suggest an independent bidirectional relationship between AD and LOE, even when accounting for the influence of mortality.

In the UCLA ATLAS genetic subsample, we further evaluated the role of AD-related genetic risk in both conditions. *APOE* was significantly associated with AD and showed a modest, borderline association with LOE, suggesting it may contribute to both outcomes. However, this association with LOE was not replicated in the All of Us validation cohort, providing limited evidence for its generalizability and likely reflecting limited power to detect small effect sizes, differences in sample size, ancestry composition, or EHR-derived phenotype accuracy between the UCLA and All of Us cohorts. These results highlight the need for further validation in well-powered, ancestrally representative datasets.

Initially, we attempted to estimate the genetic correlation between these conditions using the established LDSC tool^17^. However, results indicated no significant genetic correlation between AD and generalized epilepsy. One possible explanation is that we used a generalized epilepsy^23^ GWAS due to the lack of GWAS data specific to LOE, and the genetic risk factors may differ between these conditions. Another limitation of LDSC is that it averages the genetic effects across the entire genome^17^. When only a few loci are shared and these loci, such as the *APOE* gene, have strong effects, the signal from these specific SNPs may be diluted by the large number of non-shared SNPs with little or no effect. As a result, the overall estimate of genetic correlation may appear weaker than it actually is.

Despite the null results from LDSC, we hypothesized the existence of underlying genetic predispositions and overlapping biological pathways between AD and LOE. This led us to develop a multi-task Elastic Net model to identify these shared genetic risk factors. When selecting candidate SNPs for the multi-task Elastic Net model, we moved beyond the typical reliance on GWAS p-values and the closest gene, opting instead to prioritize Combined Annotation-Dependent Depletion (CADD) scores and functional mapping results. This strategy not only enhanced model interpretability by targeting variants with functional consequences and pinpointing likely causal loci, but also yielded significantly higher predictive accuracy compared to conventional p-value filtering^28^, allowing us to frame our findings in the broader context of genes and molecular pathways underlying shared AD and LOE risk.

Our findings provide initial evidence for potential shared genetic architecture between AD and LOE within a European population, though the extent and independence of this shared architecture require further investigation. Our multi-task Elastic Net model selected multiple shared-risk SNPs mapping to the *APOE* region, reinforcing the hypothesis that shared genetic risks exist between AD and LOE, particularly in the *APOE* region. These findings align with previous studies^18,19,29^. *APOE*, as the primary lipid carrier in the brain, is crucial for maintaining neuronal and synaptic function and repair mechanisms in the central nervous system. Dysregulation of *APOE* function, especially in the context of the *APOE-ε4* allele, may contribute to altered amyloid-beta metabolism^30,31^, neuronal hyperexcitability^32^, and neuroinflammation^33^, potentially leading to both the cognitive decline seen in AD and the increased susceptibility to seizures in LOE.

However, the identification of shared-risk SNPs outside the *APOE* region by the multi-task Elastic Net model suggests that other genetic risk factors also contribute to the shared genetics of AD and LOE. Of particular interest is the identification of the *BIN1* and *CLU* genes. *BIN1* (Bridging Integrator 1) is a major genetic risk factor for AD, known to regulate calcium homeostasis, electrical activity, and gene expression in glutamatergic neurons^34,35^. Its involvement in membrane dynamics, endocytosis, and neurotransmitter vesicle release^36^ suggests a role in synaptic function and neuronal health, which are critical for both AD pathology and seizure susceptibility. *CLU* (Clusterin), also known as apolipoprotein J, is another significant genetic risk factor for AD^37^. It is involved in amyloid-beta clearance and aggregation^38^, inflammation, and oxidative stress^39^. Given its role in maintaining neuronal integrity and responding to cellular stress, *CLU* may contribute to the shared pathogenic mechanisms underlying both AD and LOE^40^. The identification of these genes reinforces the complex genetic interplay beyond *APOE* in the shared etiology of AD and LOE.

Enrichment analysis of the identified genes enhances our understanding of the pathogenesis underlying the interconnection between these two diseases. Our analysis revealed a notable enrichment in tau protein binding with identified genes such as *BIN1*, *CLU*, and *APOE*. Tau protein, a critical microtubule-associated protein, has been implicated in the pathophysiology of epilepsy and AD, signifying its potential as a therapeutic target^35,41^. Remarkably, tau reduction has been identified as a viable therapeutic strategy for AD and other related disorders characterized by epileptic activity. In a mouse model of AD, genetic reduction of tau levels was found to prevent learning and memory disruption, epileptic activity, and other AD-related deficits^42^. Furthermore, biomarkers associated with tau, such as total tau and p-tau levels in cerebrospinal fluid^41,43,44^ or plasma^45^, are used to diagnose AD and may hold promise for LOE. This evidence, along with our results, suggests that tau-mediated mechanisms may provide a novel opportunity for the development of disease-modifying therapies for both LOE and AD.

In addition, we conducted secondary analyses to explore the utility of the predicted value derived from the multi-task Elastic Net model and validated all our findings in an independent cohort—the All of Us dataset. Our findings demonstrated the shared GRS’s capability to effectively stratify patients into distinct AD-LOE risk groups, allowing for better risk assessment and management. The consistent results across these distinct datasets underscore the reliability of our identified shared genetic risks and their potential applicability to broader populations.

On the other hand, several findings warrant careful interpretation. First, pathway enrichment analyses primarily highlighted AD-related biological processes, with minimal evidence supporting epilepsy-specific pathways, suggesting a more limited genetic overlap than initially hypothesized. Second, we observed no significant association between *APOE-ε4* and LOE in the All of Us cohort, a finding that diverges from some prior reports and calls into question the strength of *APOE*’s contribution to LOE susceptibility. Finally, associations with the shared genetic risk score appeared to be driven predominantly by AD cases, rather than showing comparable effects across both conditions. Collectively, these findings provide only limited evidence for shared genetic architecture and suggest that although some genetic overlap exists, the extent of shared genetic architecture between AD and LOE appears limited and requires further validation in larger, more diverse cohorts. Future studies should aim to replicate these findings in larger, independent cohorts and explore the functional consequences of the identified variants to better understand their role in the shared pathophysiology of AD and LOE.

While this study advances our understanding of potential shared genetic risk between AD and LOE, several limitations must be acknowledged. The prevalences of AD and LOE were lower in both our discovery and validation datasets, likely reflecting the under-diagnosis of both conditions in EHRs. Participant samples in our study were selected based on ICD-10 diagnosis codes retrieved from EHRs, which could be subject to misclassifications. For example, individuals classified with LOE could also have undiagnosed mild cognitive impairment or AD. Similarly, some diagnosed with AD could exhibit subclinical seizures. Another limitation is the study’s applicability to individuals of European ancestry only. Due to the small sample sizes of other ancestry groups in our dataset, we did not perform modeling for these populations. As a result, the generalizability of our findings to other ancestries is limited. Future studies should consider the impact of sample heterogeneity during model development. Training with larger sample sizes, collecting data from multiple institutions, and focusing on additional populations may help enhance the generalizability.

In conclusion, our study provides evidence for potential shared genetic risks of AD and LOE in the European population by deploying a multi-task learning method. While our results confirmed the importance of the *APOE* gene region and identified novel genes that may contribute to both diseases, future studies with larger sample sizes, more diverse populations, and LOE-specific GWAS data will be essential to definitively establish the extent of genetic overlap between these conditions. By highlighting these novel genes, our study contributes to a deeper understanding of the molecular mechanisms that govern the complex interplay between neurodegenerative diseases. These findings can inform future research to improve disease prevention, diagnosis, treatments, and drug development for both conditions.

## Methods

### Data source

Our primary samples were derived from the electronic health records (EHRs) of the UCLA Health System. The UCLA Health System encompasses two hospitals and 210 primary and specialty outpatient sites across greater Los Angeles. Since March 2, 2013, the UCLA Data Discovery Repository (DDR) has collected de-identified EHRs under the auspices of the UCLA Health Office of Health Informatics Analytics and the UCLA Institute for Precision Health. The DDR includes longitudinal records of demographics, vital signs, diagnoses, laboratory tests, encounters, provider metadata, prescriptions, and hospital admissions^46^. Mortality data were ascertained via in-hospital records, family reports, and California Department of Public Health death registries.

### Sample selection

To focus on longitudinal patient records, our analyses were restricted to patients with complete demographic information (age and sex) who self-reported as non-Hispanic White and had at least two medical encounters after the age of 60. An age range of 60 to 90 years was applied based on two considerations: (1) to ensure patients were old enough to meet the criteria for LOE, defined as onset after age 60; and (2) to address limitations in EHR data, where patients aged 90 or older are recorded as 90 in the UCLA de-identified EHR to maintain privacy. The final analytical sample from the full UCLA cohort comprised *N* = 416,212 patients. For the UCLA ATLAS sample, which included available genetic data, we further ensured that self-reported non-Hispanic White patients also had European American genetically inferred ancestry to enhance precision in ancestry reporting. This resulted in *N* = 16,500 patients in the UCLA ATLAS sample.

### Phenotype definition

To identify individuals with LOE or AD, we utilized a previously validated set of phecodes. Phecodes are groupings of International Classification of Diseases, Tenth Revision (ICD-10) codes that correspond to specific clinical phenotypes^47^. For LOE, the phecodes used in this study included “345” (Epilepsy, recurrent seizures, convulsions), “345.1” (Epilepsy), “345.11” (Generalized convulsive epilepsy), “345.12” (Partial epilepsy), and “345.3” (Convulsions). Individuals were classified as LOE cases if they met two criteria: (1) at least one encounter with any of these phecodes, and (2) the first LOE diagnosis occurred after age 60, with at least one encounter before age 60.

For AD, the phecode “290.11” was used, and AD cases were defined as individuals with at least one encounter with this code.

Non-LOE or non-AD controls were defined as individuals who: (1) had no diagnosis of LOE or AD, and (2) had no diagnosis of phecodes within the “exclude ranges” for each corresponding phecode. These exclude ranges were employed to mitigate inaccuracies in ICD coding, where less specific codes are sometimes applied to certain medical conditions^48^. These exclusion criteria ensured the selection of controls without related conditions, thereby providing more accurate and reliable data for EHR-based case-control analysis. For instance, phecodes “290.12” (Dementia with cerebral degenerations) and “290.16” (Vascular dementia) were excluded from the AD control group. Supplementary Table 8 provides detailed descriptions of the phecodes, exclude ranges, and mapped ICD-10 codes used in our phenotype definition.

We also collected data on other health conditions potentially associated with LOE and/or AD, including hypertension (phecode “401.1”), diabetes (phecode “250.2”), stroke (phecodes “433”), and hyperlipidemia (phecode “272.1”). All ICD-phecodes mappings were performed using the R package “*PheWAS*”^49^. Baseline characteristics were compared across LOE and AD status. χ^2^ test and analysis of variance were used as appropriate to assess homogeneity across groups.

### Genetic data pre-processing

#### Quality control

A subsample of UCLA Health patients participates in the UCLA ATLAS Community Health Initiative (ATLAS), which provides biosamples linked to their de-identified EHRs. All procedures adhered to UCLA Institutional Review Board (IRB)-approved protocols; informed consent was obtained prior to enrollment, and comprehensive biobanking details are described previously^50,51^. This study was classified as exempt human-subjects research (UCLA IRB #21–000435). For the UCLA ATLAS sample, stringent quality control (QC) measures and genotype imputation procedures were implemented to ensure the robustness and reliability of subsequent analyses. QC was conducted using PLINK v1.9^52^, following established guidelines^46^. Samples with a missingness rate exceeding 5% were excluded. Low-quality SNPs, including those with more than 5% missingness, monomorphic SNPs, and strand-ambiguous SNPs, were removed. Following QC, genotype imputation was performed using the Michigan Imputation Server^53^ to enhance the coverage of genetic variants and facilitate comparisons across different genotyping platforms. SNPs with an imputation r² below 0.90 or a minor allele frequency of less than 1% were pruned. After these procedures, approximately 8 million genotyped SNPs were retained across the 54,935 individuals.

#### Inferring genetic ancestry

Genetic ancestry refers to the most recent geographic origins of an individual’s genome, which is independent from an individual’s cultural identity^54^. Genetically inferred ancestry utilizes genetic data, reference populations, and inference methods to characterize individuals within a group who likely share recent geographical ancestors^55^. For the UCLA ATLAS sample, we employed principal component analysis (PCA)^56^ to infer patients’ genetic ancestry, using the reference panel from the 1000 Genomes Project^57^. This panel includes genotypes from individuals of known European, African, Admixed American, East Asian, and South Asian descent. For instance, “European American genetically inferred ancestry " refers to individuals in the United States whose recent biological ancestors were inferred to be of European descent. Ancestry-specific principal components (PCs) were subsequently recalculated using PCA to identify population structures within each ancestry group.

#### *APOE-ε4* count and AD polygenic risk score

The *APOE* gene has two key variants, rs7412 and rs429358, which determine the three common isoforms of the apolipoprotein E (apoE) protein: E2, E3, and E4, encoded by the ε2, ε3, and ε4 alleles, respectively^58^. To quantify the *APOE* genotype in our study, we created a numerical variable, “*APOE-ε4* count”, representing the number of ε4 alleles (0, 1, or 2) carried by each individual.

We computed the AD PRS as the sum of an individual’s risk allele dosages, each weighted by its corresponding risk allele effect size from the AD genome-wide association study (GWAS) summary statistics^15^, as shown in the PRS equation: $\:{PRS}_{i}={\sum\:}_{j}^{M}{\widehat{\beta\:}}_{j}\times\:{dosage}_{ij}$. To isolate the effect of *APOE* gene from other genetic factors, we constructed the PRS without including the *APOE* region. Specifically, for our scores with the *APOE* gene region removed, we excluded all variants from the summary statistics on chromosome 19 near *APOE* (44,891,220 to 44,919,349, GRCh38). This region spans from 10 kilobases upstream of the start position of *TOMM40* (44,891,220) to 10 kilobases downstream of the stop position of *APOC1* (44,919,349). This entire region was removed due to the dense LD block observed in European ancestries, which overlaps these three genes (*TOMM40*, *APOE*, *APOC1*).

#### Candidate single nucleotide polymorphisms (SNPs) identification and annotation

To identify shared genetic risk SNPs, we initially selected a set of genome-wide significant and potentially causal SNPs from recent GWAS on AD^24^ and epilepsy^23^. As there is currently no GWAS specifically focused on LOE, a generalized epilepsy GWAS was used as a substitute. We utilized FUMA^59^, a comprehensive tool that integrates information from various biological data repositories and resources, to annotate and prioritize these SNPs.

FUMA first identifies SNPs that have reached a genome-wide significant P-value (< 5e-8). Then it implements a sophisticated LD-based approach to identify and annotate genetic variants that extend beyond the SNPs directly tested in the original GWAS. Specifically, FUMA incorporates “proxy variants” from its reference LD panel (1000 Genomes Project by default) to comprehensively define and annotate each genomic locus^57^. The mapping from SNPs to genes was subsequently performed using ANNOVAR^60^ (“gene-based annotation”) with Ensembl genes (build 85). SNPs were mapped to genes through three distinct methods: (i) positional mapping, based on physical location on the genome; (ii) eQTL associations, through eQTL mapping; and (iii) 3D chromatin interactions, via chromatin interaction mapping. The Combined Annotation-Dependent Depletion (CADD) score^61^ was also applied to select potential causal SNPs, with higher scores indicating a greater likelihood of the variant being deleterious. SNPs with at least one positional, eQTL, or chromatin interaction mapped gene, or with a CADD score above the suggestive threshold (≥12.37), were considered deleterious.

A total of 4,454 SNPs were identified as genome-wide significant and potentially causal SNPs from the two GWAS summary statistics. These SNPs were subsequently used as candidate features for identifying shared genetic risk loci for AD and LOE.

### Statistical modeling

#### Associations between LOE and AD phenotypes

We first compared baseline demographic, comorbidities, and AD genetics measures (where applicable) across LOE and AD status in our analytical samples. Continuous variables (e.g., age) were analyzed using the Wilcoxon rank-sum test (Mann–Whitney U test), whereas categorical variables (e.g., sex) were assessed with Pearson’s chi-squared test. We then examined the associations between LOE and AD phenotypes in our analytic samples, which included both the full UCLA patient cohort (*N* = 416,212) and the ATLAS sample with available genetic data (*N* = 16,500). First, we employed Cox proportional hazard models to assess the association between the presence of LOE and the incidence of AD, and vice versa, thereby indicating the cause-specific hazard of one condition on the other. Second, we applied the Fine and Gray proportional subdistribution hazards model. This model accounts for competing risks, such as death, by retaining them in the risk set indefinitely^22^. The Fine and Gray model focuses on the cumulative incidence function, which reflects the probability of the outcome occurring over time while considering the possibility that death could preclude the occurrence of the outcome.

For all association models, demographic adjustments included age at first visit, sex, and EHR record length. Additional adjustments accounted for health risk factors associated with both conditions, including hypertension, diabetes, stroke, and hyperlipidemia status. For the genetic sample, we also adjusted for *APOE-ε4* count and AD PRS without the *APOE* region to examine the effect of genetic variables on the associations. We calculated the follow-up time for each patient, considering both the time to event and censoring. Patients with the outcome prior to the onset of exposure were excluded from the analysis. We reported hazard ratios (HR) from Cox and Fine and Gray models, along with their 95% confidence intervals (CIs).

#### Genetic correlation with linkage disequilibrium score (LDSC) regression

We evaluated the genetic correlations between AD and generalized epilepsy using the linkage disequilibrium score (LDSC) regression method with publicly available GWAS summary statistics. We utilized GWAS summary data from studies on AD^24^ and generalized epilepsy^23^, and performed genetic correlation estimation using LDSC software^62^. Briefly, LDSC software estimates the genetic correlation between two phenotypes by comparing the strength and direction of association signals at each locus while adjusting for the correlation expected due to genetic linkage^62^. We adjusted for linkage between SNPs (i.e., covariance due to genomic proximity) using LD scores based on the European sample from the 1000 Genomes Project^63^. Additional SNP QC procedures followed the defaults in the LDSC ‘*munge_sumstats.py*’ function, ensuring that alleles matched those in the HapMap3 reference panel^64^. The genetic correlation was assessed using the intersection of QC-positive SNPs, and results were reported as the correlation coefficient ± standard error.

#### Identification of shared risk genetic loci of AD and LOE with multi-task learning

We employed a multi-task learning framework to identify shared genetic risk loci of AD and LOE in a separate UCLA ATLAS sample. The modeling sample (*N* = 9,986) was further restricted by applying stricter inclusion criteria to controls: age at last visit ≥ 70 and a minimum of five years of records. These criteria reduced the potential bias from misdiagnosis. Given the limited number of cases for both phenotypes (658 cases for LOE and 376 cases for AD), reducing the number of controls also helped mitigate bias due to the imbalanced dataset^65^.

To distinctly assess genetic influences, we began by mitigating the impact of demographic factors, including age, sex, and ancestry-specific PCs. We first employed a logistic regression model using these variables to predict AD or LOE status, deriving predicted values for each patient. Using a logit inverse link function, we subtracted these predicted values from the observed outcomes (AD or LOE status), generating “offset” values. These offsets represented the AD or LOE status after regressing out the effects of demographic variables and genetic population structure.

Next, we trained a multi-task Elastic Net model to predict AD and LOE status, applying offset corrections in the linearized space, expressed as: $\:{\widehat{y}}_{i}={g}^{-1}({\beta\:}_{0}+{\beta\:}_{1}{x}_{i1}+\dots\:+{\beta\:}_{p}{x}_{ip}+{offset}_{i})$, where $\:{\widehat{y}}_{i}$ represents the predicted dementia status, and $\:{g}^{-1}\left(\right)$ is the inverse of the link function^66^.

Figure 2 illustrates the multi-task Elastic Net model. In general, a multi-task learning model is trained simultaneously on multiple related tasks, leveraging shared information to improve performance across all tasks. The multi-task Elastic Net model consists of a linear model trained with a mixed ℓ1 ℓ2-norm and ℓ2-norm for regularization, using coordinate descent to fit the coefficients^67^.

The multi-task Elastic Net model also employs Elastic Net regularization, which combines the advantages of both Lasso (L1) and Ridge (L2) regression methods. This approach enhances model stability and variance handling, aiding in variable selection by reducing the coefficients of less relevant variables to zero, thereby simplifying the model and improving its ability to manage multicollinearity—particularly useful given the many SNP features in linkage disequilibrium (LD)^68^. The hyperparameter α, which controls the strength of L1 regularization, and $\:\rho\:$, which balances L1 and L2 regularization, were optimized using a grid search to maximize the penalized likelihood within the training set.

To enhance the robustness of our findings, we repeated the modeling process 1,000 times with different random seeds to determine feature importance and establish statistical significance. Model performance and features selected by the multi-task Elastic Net model in each iteration were recorded. SNPs identified in at least 25% of the 1,000 iterations were retained. Shared genetic risk loci for AD and LOE were defined as features with average coefficients in the same direction for both tasks across all iterations.

For biological interpretations, we mapped identified risk SNPs to genes using FUMA’s positional, eQTL, and chromatin interaction mapping^59^. Specifically, we used: (1) positional mapping based on genomic coordinates from the GRCh38 reference genome, mapping SNPs to genes within ± 100 kb windows; (2) eQTL mapping using data from the GTEx v8 database, focusing on brain-relevant tissues including cortex, hippocampus, and other neural tissues; and (3) chromatin interaction mapping using Hi-C data from the 3D Genome Browser and published chromatin conformation capture studies in neural cell types. Functional consequences of each variant were predicted using the Ensembl Variant Effect Predictor (VEP) database, which integrates multiple prediction algorithms including SIFT, PolyPhen-2, and CADD scores^69^. Genes of interest (e.g., mapped shared risk genes of AD and LOE) were tested for enrichment of biological functions using hypergeometric tests against gene sets from MsigDB. Gene sets with an adjusted P-value ≤ 0.05 and more than one overlapping gene were reported and visualized using heatmaps.

### Sensitivity analyses

#### Shared AD-LOE genetic risk score on AD and LOE diagnosis

We constructed an AD-LOE shared genetic risk score (GRS) for each patient using the shared risk factors identified in prior analyses. The full UCLA ATLAS sample was randomly split into a training set (80%) and a testing set (20%). For each individual $\:i$, the shared GRS was calculated as: $\:shared\_GR{S}_{i}=\sum\:_{j}^{M}{\widehat{\beta\:}}_{j}\times\:dosag{e}_{ij}$, where $\:M$ represents the total number of shared SNPs, and $\:{\widehat{\beta\:}}_{j}$ represents the estimated weight for a given shared risk SNP $\:j$. The $\:{\widehat{\beta\:}}_{j}$ for each SNP was determined by fitting a logistic regression model on the training set (*N* = 13,200), using all identified shared risk SNPs as predictors and AD or LOE as the outcome. Consistent with earlier modeling steps, we first regressed out the effects of demographic variables and genetic population structure. We standardized the final shared GRS to a mean of 0 and a standard deviation of 1 (defined by the training set) to reflect per-unit increases in the score.

The resulting shared GRS was standardized (mean = 0, SD = 1) based on the training set to facilitate interpretation per unit increase. Associations between the standardized shared GRS and AD or LOE status were subsequently evaluated in the independent testing set (*N* = 3,300) using logistic regression models, adjusting for sex and the first five ancestry-specific PCs. ORs and their 95% CIs were reported.

Based on the logistic regression models, we also calculated the variance explained by the AD-LOE shared GRS for each phenotype. A reduced logistic regression model was fitted with sex and the first five ancestry-specific PCs only. The percentage of variation explained by the AD-LOE shared GRS was calculated using the formula:

$$\:Explained\:variance\:\left(\%\right)=100\times\:\frac{{deviance}_{reduced\:model}-{deviance}_{full\:model}}{{deviance}_{reduced\:model}}$$

Given that the *APOE* gene is a well-studied shared risk factor between AD and LOE, we also compared the variance explained by our AD-LOE shared GRS to that explained by the *APOE-ε4* allele.

To address potential concerns about the dominance of *APOE* region variants in our shared genetic risk score and to evaluate the independent contribution of non-*APOE* variants to shared genetic risk, we conducted a sensitivity analysis using only the four shared risk variants located outside the *APOE* region. These variants included chr2:127133851 (*BIN1*), chr8:27607412 (*CLU*), chr10:11676714 (*PVRL2*), and chr2:103489151 (*TRAPPC6A*). The non-*APOE* shared genetic risk score was constructed using identical methodology as the full shared GRS described above.

#### Model evaluation for AD or LOE prediction

As part of our sensitivity analysis, we evaluated the performance of our multi-task Elastic Net model in predicting AD and LOE phenotypes by comparing it with a separate Elastic Net model that predicts AD and LOE individually. The primary assessment criterion was the Area Under the Precision-Recall Curve, chosen for its effectiveness in scenarios involving imbalanced datasets, where the number of cases significantly outnumbers the controls^70^. The same 1,000 iteration steps were applied, and results were reported on the testing set of each iteration. To compare the models, we used the paired Wilcoxon signed-rank test^71^, as this test is suitable for comparing two metrics derived from the same observations.

#### Validation in All of Us dataset

To further assess robustness, we validated our results in the All of Us Research Program, a geographically and demographically diverse U.S. cohort of over 500,000 participants^72^. We employed Data Release v8, which contains longitudinal clinical data for 393,596 individuals through October 1, 2023. All analyses were performed within the All of Us Researcher Workbench under approved data-use protocols, and we derived a comparable subset from the All of Us Research Hub using the same inclusion criteria.

First, we repeated the same association tests of AD and LOE with the Cox proportional hazard models and the Fine and Gray models in the All of Us sample. The same variables were adjusted in these models. Next, we applied the GRS weights trained from the UCLA ATLAS sample to the All of Us sample and constructed an AD-LOE shared GRS for each patient in the All of Us cohort. Similarly, we tested the associations between the shared AD-LOE GRS and both AD and LOE in this sample and compared them to the *APOE-ε4* model.

We considered p-values < 0.05 as statistically significant unless otherwise specified. Codes used to produce all analyses in this manuscript are available online (https://github.com/TSChang-Lab/AD_LOE_shared_genetics).

## Supplementary Information

Below is the link to the electronic supplementary material.

Supplementary Material 1

## Data Availability

Individual electronic health record data are not publicly available due to patient confidentiality and security concerns. Collaboration with the study authors who have been approved by UCLA Health for Institutional Review Board-qualified studies are possible and encouraged. Code is available on GitHub: https://github.com/TSChang-Lab/AD_LOE_shared_genetics.
