# Supplementary material for: Multi-task learning identifies shared genetic risk for late-onset epilepsy and alzheimer’s disease
Source: Sci Rep. 2025 Dec 13;16:2517. doi: 10.1038/s41598-025-32329-8 (PMC12820071; doi:10.1038/s41598-025-32329-8)
Supplement: Supplementary file 1 — Supplementary Material 1 [file 41598_2025_32329_MOESM1_ESM.pdf]

Supplementary Table 1. Descriptive statistics of the analytical sample, UCLA patient with (ATLAS) vs. without genetic data (non-ATLAS)[a]

	**Overall N = 416,212**	**ATLAS N = 16,500**	**Non-ATLAS N = 399,712**	**P-value**
**Primary outcome(s)**				
Alzheimer's disease (Yes)	6,706 (1.6%)	376 (2.3%)	6,330 (1.6%)	<0.001*
Late onset epilepsy (Yes)	7,351 (1.8%)	658 (4.0%)	6,693 (1.7%)	<0.001*
**Demographics**				
Age at first visit (in years)	60.12 (11.33)	56.23 (11.56)	60.28 (11.29)	<0.001*
Age at last visit (in years)	72.37 (7.87)	73.36 (7.57)	72.32 (7.88)	<0.001*
Sex (female)	225,008 (54%)	8,092 (49%)	216,916 (54%)	<0.001*
EHR record length (in years)	12.25 (9.86)	17.14 (10.22)	12.05 (9.79)	<0.001*
Deceased (Yes)	19,727 (4.7%)	1,362 (8.3%)	18,365 (4.6%)	<0.001*
**Health conditions**				
Hypertension (Yes)	148,074 (36%)	11,375 (69%)	136,699 (34%)	<0.001*
Diabetes (Yes)	45,016 (11%)	3,704 (22%)	41,312 (10%)	<0.001*
Stroke (Yes)	2,840 (0.7%)	363 (2.2%)	2,477 (0.6%)	<0.001*
Hyperlipidemia (Yes)	126,312 (30%)	12,113 (73%)	114,199 (29%)	<0.001*

Abbreviations: EHR, electronic health record.

Notes:

[a] Summary statistics are displayed as n (%) for categorical variables, and mean (SD) for continuous variables. P-values were calculated with Pearson's Chi-squared test or Wilcoxon rank sum test as appropriate.

* Statistical significance at P-value < 0.05 level.

Supplementary Table 2. Associations between Alzheimer's disease (AD) and late-onset epilepsy (LOE), UCLA ATLAS patient population (N = 16,500)[a]

		***APOE-ε4* count**		**AD PRS without *APOE* region**	
**AD ~ LOE**	**N**	**HR [95% CI]**	**P-value**	**HR [95% CI]**	**P-value**
Cox proportional hazard	16,480	2.53 (2.10, 3.05)	<0.001*	1.02 (0.93, 1.12)	0.64
Fine and Gray	16,480	2.51 (2.07, 3.05)	<0.001*	1.02 (0.93, 1.12)	0.62
**LOE ~ AD**					
Cox proportional hazard	16,462	1.10 (0.93, 1.30)	0.29	1.01 (0.94, 1.08)	0.84
Fine and Gray	16,462	1.08 (0.91, 1.28)	0.36	1.01 (0.94, 1.09)	0.73

Abbreviations: AD, Alzheimer's disease; CI, confidence interval; EHR, electronic health record; HR, hazard ratio.

Notes:

[a] Basic Model adjustments include age at first visit, sex, EHR record length, hypertension, diabetes, stroke, and hyperlipidemia status.

Supplementary Table 3. Descriptive statistics of the analytical sample by late-onset epilepsy and Alzheimer's disease (AD) status, multi-task Elastic Net modeling sample (subset of UCLA ATLAS patient) (N = 9,986)[a]

	Late-onset epilepsy			Alzheimer's disease		
	**Cases N = 658**	**Controls N = 9,328**	**P-value**	**Cases N = 376**	**Controls N = 9,610**	**P-value**
**Primary outcome(s)**						
Alzheimer's disease (Yes)	44 (6.7%)	332 (3.6%)	0.001*	376 (100%)	0 (0%)	-
Late onset epilepsy (Yes)	658 (100%)	0(0%)	-	44 (12%)	614 (6.4%)	<0.001*
**Demographics**						
Age at first visit (in years)	58.19 (10.36)	58.74 (10.82)	0.04*	62.08 (11.83)	58.57 (10.73)	<0.001*
Age at last visit (in years)	75.95 (6.77)	77.97 (5.25)	<0.001*	82.01 (5.93)	77.68 (5.29)	<0.001*
Sex (female)	298 (45%)	4,566 (49%)	0.07	207 (55%)	4,657 (48%)	0.01*
EHR record length (in years)	17.76 (9.81)	19.24 (10.23)	0.002*	19.93 (10.92)	19.11 (10.18)	0.1
Deceased (Yes)	141 (21%)	813 (8.7%)	<0.001*	67 (18%)	887 (9.2%)	<0.001*
**Health conditions**						
Hypertension (Yes)	526 (80%)	7,140 (77%)	0.046*	296 (79%)	7,370 (77%)	0.4
Diabetes (Yes)	188 (29%)	2,344 (25%)	0.05	103 (27%)	2,429 (25%)	0.4
Stroke (Yes)	48 (7.3%)	274 (2.9%)	<0.001*	54 (14%)	268 (2.8%)	<0.001*
Hyperlipidemia (Yes)	524 (80%)	7,514 (81%)	0.6	304 (81%)	7,734 (80%)	0.9

Abbreviations: EHR, electronic health record.

Notes:

[a] Summary statistics are displayed as n (%) for categorical variables, and mean (SD) for continuous variables. P-values were calculated with Pearson's Chi-squared test or Wilcoxon rank sum test as appropriate.

* Statistical significance at P-value < 0.05 level.

Supplementary Table 4. Full shared risk genetic loci (same direction) identified from multi-task Elastic Net model (prediction of either Alzheimer's disease or late-onse epilepsy), based on 1000 iteration results, UCLA ATLAS patient population (N = 9,986)

No.	rsID	CHR	POS	REF	ALT	Nearest Gene	Function	Phenotype	Position Map	eQTL Map	CI Map	Trained GRS weight
1	rs283811	19	44885243	A	G	*PVRL2:CTB-129P6.4*	ncRNA_intronic	AD	1	0	0	0.0211
2	rs28399637	19	44820881	G	A	*BCAM*	intronic	AD	1	0	0	0.0116
3	rs4663105	2	127133851	A	C	*BIN1*	intergenic	AD	0	1	0	0.0126
4	rs13395153	2	103489151	T	C	*AC092568.1*	intergenic	Epilepsy	1	0	0	-0.0097
5	rs3925681	19	44917843	G	A	*APOC1*	intronic	AD	1	1	1	-0.0050
6	rs7912495	10	11676714	A	G	*RP11-138I18.2*	intergenic	AD	1	0	0	0.0122
7	rs4236673	8	27607412	G	A	*CLU*	intronic	AD	1	0	1	-0.0155
8	rs7254133	19	44935297	C	T	*APOC1P1*	intergenic	AD	1	0	1	0.0100

Abbreviations: AD, Alzheimer's Disease; CI, chromatin interaction; eQTL, expression quantitative trait loci; GRS, Genetic Risk Score.

Supplementary Table 5. Mapped genes of selected risk genetic loci (same direction) identified from multi-task Elastic Net model (prediction of either Alzheimer's disease or late-onset epilepsy), based on 1000 iteration results, UCLA ATLAS patient population (N = 9,986)

No.	Gene	Symbol	CHR	Start	End	Type	posMap	eqtlMap	ciMap	Phenotype
1	ENSG00000236596	*AC092568.1*	2	104110699	104112300	pseudogene	Yes	No	No	LOE
2	ENSG00000136717	*BIN1*	2	127805603	127864931	protein_coding	Yes	Yes	No	AD
3	ENSG00000120885	*CLU*	8	27454434	27472548	protein_coding	Yes	No	No	AD
4	ENSG00000271046	*RP11-138I18.2*	10	11721674	11722513	lincRNA	Yes	No	No	AD
5	ENSG00000062370	*ZNF112*	19	44830708	44871377	protein_coding	Yes	Yes	No	AD
6	ENSG00000187244	*BCAM*	19	45312328	45324673	protein_coding	Yes	Yes	No	AD
7	ENSG00000130202	*PVRL2*	19	45349432	45392485	protein_coding	Yes	Yes	No	AD
8	ENSG00000267282	*CTB-129P6.4*	19	45385284	45394133	antisense	Yes	No	Yes	AD
9	ENSG00000130204	*TOMM40*	19	45393826	45406946	protein_coding	Yes	No	Yes	AD
10	ENSG00000130203	*APOE*	19	45409011	45412650	protein_coding	Yes	No	No	AD
11	ENSG00000130208	*APOC1*	19	45417504	45422606	protein_coding	Yes	Yes	Yes	AD
12	ENSG00000214855	*APOC1P1*	19	45430061	45434643	pseudogene	Yes	Yes	Yes	AD
13	ENSG00000224916	*APOC4-APOC2*	19	45445495	45452822	protein_coding	Yes	No	No	AD
14	ENSG00000267467	*APOC4*	19	45445495	45452820	protein_coding	Yes	Yes	No	AD
15	ENSG00000267114	*CTB-129P6.11*	19	45453301	45457264	protein_coding	Yes	No	Yes	AD
16	ENSG00000104853	*CLPTM1*	19	45457842	45496599	protein_coding	Yes	Yes	Yes	AD
17	ENSG00000104859	*CLASRP*	19	45542298	45574214	protein_coding	Yes	No	Yes	AD
18	ENSG00000007255	*TRAPPC6A*	19	45666186	45681495	protein_coding	Yes	Yes	Yes	AD
19	ENSG00000189114	*BLOC1S3*	19	45682003	45685059	protein_coding	Yes	Yes	Yes	AD
20	ENSG00000267545	*AC005779.2*	19	45683080	45705702	protein_coding	Yes	No	Yes	AD
21	ENSG00000225157	*AC005779.1*	19	45687584	45688190	pseudogene	Yes	No	Yes	AD

Abbreviaitions: AD, Alzheimer's disease; ciMap, chromatin interaction mapping; CHR, chromosome; eqtlMap, eQTL mapping; LOE, late-onset epilepsy; POS, position; posMap, positional mapping.

Supplementary Table 6. Descriptive statistics of the analytical sample, All of Us vs. UCLA ATLAS patient population[a]

	**All of Us N = 52,493**	**ATLAS N = 16,500**	**P-value**
**Primary outcome(s)**			
Alzheimer's disease (Yes)	517 (1.0%)	376 (2.3%)	<0.001*
Late onset epilepsy (Yes)	2,182 (4.2%)	658 (4.0%)	0.3
**Demographics**			
Age at first visit (in years)	63.87 (8.28)	56.23 (11.56)	<0.001*
Age at last visit (in years)	71.43 (6.69)	73.36 (7.57)	<0.001*
Sex (female)	28,894 (55%)	8,092 (49%)	<0.001*
EHR record length (in years)	7.57 (5.54)	17.14 (10.22)	<0.001*
Deceased (Yes)	810 (1.5%)	1,362 (8.3%)	<0.001*
**Health conditions**			
Hypertension (Yes)	31,697 (60%)	11,375 (69%)	<0.001*
Diabetes (Yes)	10,701 (20%)	3,704 (22%)	<0.001*
Stroke (Yes)	460 (0.9%)	363 (2.2%)	<0.001*
Hyperlipidemia (Yes)	29,133 (55%)	12,113 (73%)	<0.001*
**Alzheimer's disease genetics**			
APOE-ε4 count			<0.001*
0	44,301 (84%)	12,415 (75%)	
1	7,828 (15%)	3,874 (23%)	
2	364 (0.7%)	211 (1.3%)	

Abbreviations: APOE, apolipoprotein E; EHR, electronic health record.

Notes:

[a] Summary statistics are displayed as n (%) for categorical variables, and mean (SD) for continuous variables. P-values were calculated with Pearson's Chi-squared test or Wilcoxon rank sum test as appropriate.

* Statistical significance at P-value < 0.05 level.

Supplementary Table 7. Descriptive statistics of the analytical sample by late-onset epilepsy and Alzheimer's disease (AD) status, All of Us patient population (N = 52,493)[a]

	Late-onset epilepsy			Alzheimer's disease		
	Cases N = 2,182	Controls N = 50,311	P-value	Cases N = 517	Controls N = 51,976	P-value
**Primary outcome(s)**						
Alzheimer's disease (Yes)	58 (2.7%)	459 (0.9%)	<0.001*	517 (100%)	0 (0%)	-
Late onset epilepsy (Yes)	2,182 (100%)	0(0%)	-	58 (11%)	2,124 (4.1%)	<0.001*
**Demographics**						
Age at first visit (in years)	64.26 (7.95)	63.85 (8.30)	0.04*	68.78 (8.18)	63.82 (8.27)	<0.001*
Age at last visit (in years)	72.80 (6.52)	71.37 (6.69)	<0.001*	77.57 (7.01)	71.37 (6.65)	<0.001*
Sex (female)	1,155 (53%)	27,739 (55%)	0.04*	244 (47%)	28,650 (55%)	<0.001*
EHR record length (in years)	8.54 (5.56)	7.53 (5.54)	<0.001*	8.79 (5.44)	7.56 (5.54)	<0.001*
Deceased (Yes)	145 (6.6%)	665 (1.3%)	<0.001*	36 (7.0%)	774 (1.5%)	<0.001*
**Health conditions**						
Hypertension (Yes)	1,741 (80%)	29,956 (60%)	<0.001*	389 (75%)	31,308 (60%)	<0.001*
Diabetes (Yes)	742 (34%)	9,959 (20%)	<0.001*	158 (31%)	10,543 (20%)	<0.001*
Stroke (Yes)	134 (6.1%)	326 (0.6%)	<0.001*	32 (6.2%)	428 (0.8%)	<0.001*
Hyperlipidemia (Yes)	1,559 (71%)	27,574 (55%)	<0.001*	361 (70%)	28,772 (55%)	<0.001*

Abbreviations: EHR, electronic health record.

Notes:

[a] Summary statistics are displayed as n (%) for categorical variables, and mean (SD) for continuous variables. P-values were calculated with Pearson's Chi-squared test or Wilcoxon rank sum test as appropriate.

* Statistical significance at P-value < 0.05 level.

Supplementary Table 8. Definition of phenotypes using phecodes, related exclude ranges, and mapped ICD-10 codes

Phenotype	Phecode	Description	Exclude ranges	Mapped ICD-10
Epilepsy	345	Epilepsy, recurrent seizures, convulsions	330-337.99, 341-349.99	G40, G40.9, G41.8
	345.1	Epilepsy		G40.1, G40.9, G41, G41.0, G41.1, G41.9
	345.11	Generalized convulsive epilepsy		G40.3, G40.4
	345.12	Partial epilepsy		G40.0, G40.2
	345.3	Convulsions		G40.4, G40.5, G40.6, G40.7, G41.2, R56, R56.0, R56.8
Alzheimer's disease	290.11	Alzheimer's disease	290-292.99	F00.0, F00.1, F00.2, G30, G30.0, G30.1, G30.8, G30.9
